# Rasagiline as Adjunct to Levodopa for Treatment of Parkinson's Disease: A Systematic Review and Meta-Analysis

**DOI:** 10.1155/2022/4216452

**Published:** 2022-08-30

**Authors:** Osamu Kano, Hiroshi Tsuda, Ayako Hayashi, Masaki Arai

**Affiliations:** ^1^Department of Neurology, Toho University Faculty of Medicine, Tokyo, Japan; ^2^Department of Neurology, Juntendo University, Tokyo, Japan; ^3^Takeda Pharmaceutical Company Limited, Tokyo, Japan

## Abstract

**Background:**

Rasagiline is a selective, irreversible monoamine oxidase type B inhibitor used as monotherapy in early Parkinson's disease and as an adjunct therapy to levodopa in Parkinson's disease with motor fluctuations.

**Objectives:**

This meta-analysis aimed to provide updated evidence on the efficacy for motor and nonmotor symptoms and the safety of rasagiline/levodopa versus levodopa in patients with Parkinson's disease experiencing motor fluctuations.

**Methods:**

A systematic literature search was conducted (January 18-19, 2021) using PubMed, Cochrane Library, EMBASE, Web of Science, and Google Scholar to identify randomized controlled trials comparing rasagiline/levodopa versus placebo/levodopa in patients with Parkinson's disease experiencing motor fluctuations. Outcomes included change in wearing-off time, Unified Parkinson's Disease Rating Scale (UPDRS)/Movement Disorder Society-UPDRS (MDS-UPDRS) II and III scores, treatment-emergent adverse events (TEAEs), and Parkinson's Disease Questionnaire (PDQ-39) summary index score. A random effect model was used to estimate the treatment effects.

**Results:**

Six studies were included (1912 patients). Significant improvements in wearing-off time (standardized mean difference [SMD]: −0.50, 95% confidence interval [CI]: –0.92 to –0.09, *p* = 0.002), levodopa dosage (SMD: −0.18, 95% CI: −0.35 to –0.01, *p* = 0.041), UPDRS/MDS-UPDRS II (SMD: −0.39, 95% CI: −0.52 to –0.25, *p* < 0.0001), UPDRS/MDS-UPDRS III (SMD: −0.30, 95% CI: −0.44 to –0.16, *p* < 0.0001), and PDQ-39 summary index score (SMD: –0.21, 95% CI: –0.37 to –0.04, *p* = 0.013) were observed with rasagiline/levodopa versus placebo/levodopa. The incidence of TEAEs did not differ between treatments (risk ratio: 1.13, 95% CI: 0.98–1.30, *p* = 0.093).

**Conclusions:**

This meta-analysis further indicated the superiority of rasagiline/levodopa in improving motor and nonmotor symptoms of Parkinson's disease, with a similar safety profile to that of levodopa in Parkinson's disease with motor fluctuations.

## 1. Introduction

Parkinson's disease (PD) is a progressive neurogenerative disorder characterized by the dysfunction or loss of substantia nigra dopaminergic neurons. PD causes motor symptoms, including bradykinesia, rigidity, and resting tremor, and nonmotor symptoms, including anxiety, depression, constipation, and insomnia [1, 2]. With the aging population in Japan, the prevalence of PD has been increasing [3]; in 2016, PD had affected over 256,000 patients, and it is estimated to have affected 344,000 patients in 2020 [4, 5]. Current PD treatments primarily aim to improve motor symptoms by restoring the dopamine levels in the striatum [6, 7]. Levodopa has been the most effective treatment for improving motor dysfunction and is recommended in all stages of PD [6, 8]. However, as the disease progresses, the effect of levodopa declines [9]. The therapeutic window of levodopa narrows and each dose of levodopa lasts for a shorter duration, resulting in motor fluctuations, including “wearing-off” and dyskinesia [8–10]. Motor fluctuations and dyskinesia develop in 40–50% of patients within 5 years and 70–80% of patients within 10 years of levodopa treatment [7], causing management of PD to be challenging.

Rasagiline is a selective, irreversible, monoamine oxidase B inhibitor [11] that was approved in Europe in 2005 [12], in the US in 2006 [13], and most recently in Japan in 2018 [14], as a monotherapy in early PD and as an adjunct therapy to levodopa in PD with motor fluctuations. Rasagiline has been shown to be well tolerated and effective in improving Unified Parkinson's Disease Rating Scale (UPDRS) score [15–18], which was revised in 2008 to the Movement Disorder Society-UPDRS (MDS-UPDRS) to allow better evaluation of the nonmotor and motor experiences of daily living aspects of PD [19, 20]. Since the revision, more studies have been using the revised scale to assess the treatment effect of rasagiline [21–24]. However, systematic reviews and meta-analyses that integrated the results of these two scales are not yet available. Additionally, patients' health-related quality of life (QOL) is affected by both motor and nonmotor symptoms and therefore is an important outcome to assess in PD [25]. The Parkinson's Disease Questionnaire (PDQ-39), a PD-specific, patient-reported questionnaire, has been available to measure eight dimensions of health-related QOL, including activities of daily living, emotional well-being, and communication [26, 27]. However, almost none of the previous systematic reviews or meta-analyses have analyzed the efficacy of rasagiline/levodopa combination therapy on patient-reported outcomes, including PDQ-39.

The objective of this meta-analysis was to provide updated evidence on the safety and efficacy of rasagiline as adjunct therapy on motor and nonmotor symptoms, assessed by UPDRS and MDS-UPDRS, as well as health-related QOL such as PDQ-39, in levodopa-treated patients with PD experiencing motor fluctuations.

## 2. Methods

This systematic review and meta-analysis complied with the guidelines of Preferred Reporting Items for Systematic Reviews and Meta-Analyses (PRISMA) (see PRISMA 2020 checklist) and followed a prepared protocol (not registered).

### 2.1. Data Sources and Search Terms

All studies that compared rasagiline plus levodopa versus levodopa monotherapy were identified by searching PubMed, Cochrane Library, EMBASE, Web of Science, and Google Scholar on January 18 or 19, 2021. Search strategies were adapted to each database (Table S1) and included search terms related to the following concepts: Parkinson, Rasagiline, Levodopa. Google Scholar was used as a supplemental literature source to search for further publications that may not have been identified by searches of the other databases. Several combinations of keywords, including Parkinson, rasagiline, and levodopa, were also used, and third-party reviewers inspected the first 100 results that appeared in each search. Literature searches were restricted to English publications with accessible abstracts and full text, and duplicate publications were excluded. There were no geographic limitations.

### 2.2. Eligibility Criteria

The inclusion criteria were as follows: randomized controlled trial (RCT) of patients with PD who are experiencing motor fluctuations (including wearing-off phenomena) and treated with levodopa; compared rasagiline 1 mg/day plus levodopa versus placebo plus levodopa; and reported change in wearing-off time, change in levodopa dosage, change in UPDRS/MDS-UPDRS II, change in UPDRS/MDS-UPDRS III, change in UPDRS/MDS-UPDRS II+III, treatment-emergent adverse events (TEAEs), and change in PDQ-39 summary index score. The exclusion criteria were as follows: studies of patients with no current levodopa treatment; studies of patients receiving rasagiline 2 mg/day and/or Stalevo® (levodopa, carbidopa hydrate, entacapone) or fixed-dose combination of levodopa and entacapone; quasirandomized trials, cross-over trials, and case reports; and unpublished literature, such as conference presentations.

### 2.3. Screening and Data Extraction

From the title and abstract of each publication collected, an initial screening was conducted by two third-party reviewers independently to identify potential publications for inclusion. A secondary screening was then conducted by the two reviewers independently using the full text to assess whether the publications met the eligibility criteria. The reviewers' decision for inclusion was cross-checked, and any disagreements were resolved by considering the opinion of a third reviewer. The final list of the publications identified for inclusion was reviewed and approved by the authors. Data were extracted into qualitative tables independently by the two reviewers. Data extracted included study characteristics, patient characteristics, diagnostic criteria for PD, drug dose, and outcomes. For continuous outcomes, mean and standard deviation (SD) were collected; if these data were missing, the study was excluded from the respective analysis.

### 2.4. Outcomes

The primary outcomes were changed from baseline in wearing-off time, levodopa dosage, UPDRS/MDS-UPDRS III, and TEAEs. The secondary outcomes were changed from baseline in UPDRS/MDS-UPDRS II + III, UPDRS/MDS-UPDRS II, and PDQ-39 summary index score.

### 2.5. Assessment of Study Quality

The risk of bias in each study was assessed independently by the two reviewers by evaluating the five bias domains (randomization, intervention, missing data, measurement of the outcomes, and reported results) of the Cochrane Risk of Bias 2 tool [28]. Any disagreements regarding the level of bias risk were resolved by consensus between the two reviewers or by a third reviewer.

### 2.6. Statistical Analysis

To estimate the treatment effects, risk ratios (RRs) and their 95% confidence intervals (CIs) were used for binary or dichotomous outcomes, and standardized mean differences (SMDs) and mean differences (MDs) and their 95% CIs were used for continuous outcomes. The SMD was calculated using the Hedges' *g* method [29]; the difference in wearing-off time, levodopa dosage, UPDRS/MDS-UPDRS scores, and PDQ-39 summary index score between the rasagiline/levodopa and placebo/levodopa groups in each study was divided by the SD to standardize the results from different studies. For UPDRS/MDS-UPDRS, the pooled estimate was converted to the original scale (MDS-UPDRS) where necessary. The MD was calculated as a reference value by backtransforming the SMD (i.e., multiplying the SMD by the pooled SD of baseline scores from the most representative study selected [29]), which was the study with the largest number of patients for each efficacy outcome (wearing-off time [18], levodopa dosage [17], UPDRS/MDS-UPDRS II [18], UPDRS/MDS-UPDRS III [18], and PDQ-39 [30]). A random effect model was used to account for the differences between studies selected for estimating the related intervention effects; a *p*-value of <0.05 was considered statistically significant. Restricted maximum likelihood was used to estimate heteroskedasticity variance parameters. An I^2^ test and Q-statistics were used to assess statistical heterogeneity across studies. An I^2^ of <25%, >50%, and >75% indicates low, moderate to high, and extreme heterogeneity, respectively. The Q-statistic was defined as the weighted sum of the squared deviations of the estimates of all studies; heterogeneity was statistically significant if *p* < 0.10. Furthermore, sensitivity analysis was performed to further assess the effect of a single study by excluding studies with a high risk of bias one at a time; a two-sided *p*-value of <0.05 was considered statistically significant. Evaluation of publication bias was completed using a funnel plot, Begg's rank correlation test, and Egger's linear regression test, and a *p*-value of >0.1 was considered to indicate no publication bias. For analyses with <10 studies, a funnel plot was constructed only for reference. Statistical analyses were performed using the metafor and forest plot packages in *R* (version 4.0.2).

## 3. Results

### 3.1. Search Results and Study Characteristics

**Figure 1:**
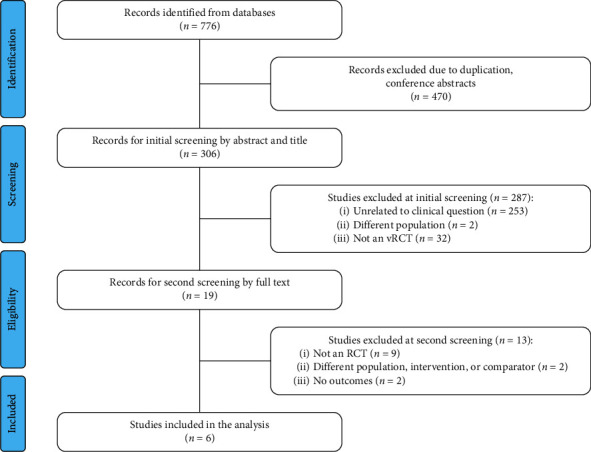
PRISMA flow diagram. PRISMA, Preferred Reporting Items for Systematic Reviews and Meta-Analyses; RCT, randomized controlled trial.

A total of 776 publications were identified from the database search (Figure 1). After removing duplicate and unpublished literature (e.g., conference abstracts), an initial screening of titles and abstracts was conducted on 306 publications. Of these, 19 full text articles were screened for their eligibility. A total of six articles met all eligibility criteria and were included in the analysis [17, 18, 24, 30–32].

The six studies included a total of 1912 patients from various countries (Europe, India, Latin America, North America, Turkey, Israel, South Africa, China, Japan). There were 945 and 967 patients who received rasagiline/levodopa and placebo/levodopa, respectively. Mean age of patients ranged from 61.6 to 66.3 years (Table 1). Mean/median baseline levodopa dosages varied widely, ranging from 399.3 mg to 821.0 mg, with the Japanese study [24] reporting the lowest mean baseline levodopa dosages compared with other studies. Mean PD duration also varied, ranging between 5.4 and 9.7 years.

All six studies reported change in wearing-off time and TEAEs, five studies reported UPDRS/MDS-UPDRS III, and four studies reported UPDRS/MDS-UPDRS II. There were two studies each that reported a change in levodopa dosage and PDQ-39 summary index score. No study reported UPDRS/MDS-UPDRS II + III; therefore, this outcome was not analyzed.

### 3.2. Risk of Bias

**Figure 2:**
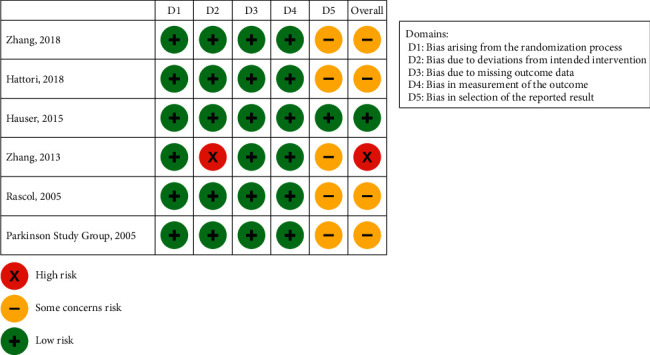
Summary of risk of bias. D, domain.

Of the six studies, one study [32] was considered to have “low risk” of bias (Figure 2). Four studies [17, 18, 24, 30] were considered to have “some concerns” related to bias because the protocols were not available, and therefore the full details of the analyses could not be assessed. One study was considered to have an overall “high risk” of bias [31]. For this study, there was “high risk” regarding possible intervention bias for not using intention-to-treat (ITT) analysis and “some concerns” regarding possible reporting bias because the protocol was not available to assess the full details of the study.

### 3.3. Efficacy

#### 3.3.1. Treatment Effect of Rasagiline on Wearing-Off Time

**Figure 3:**
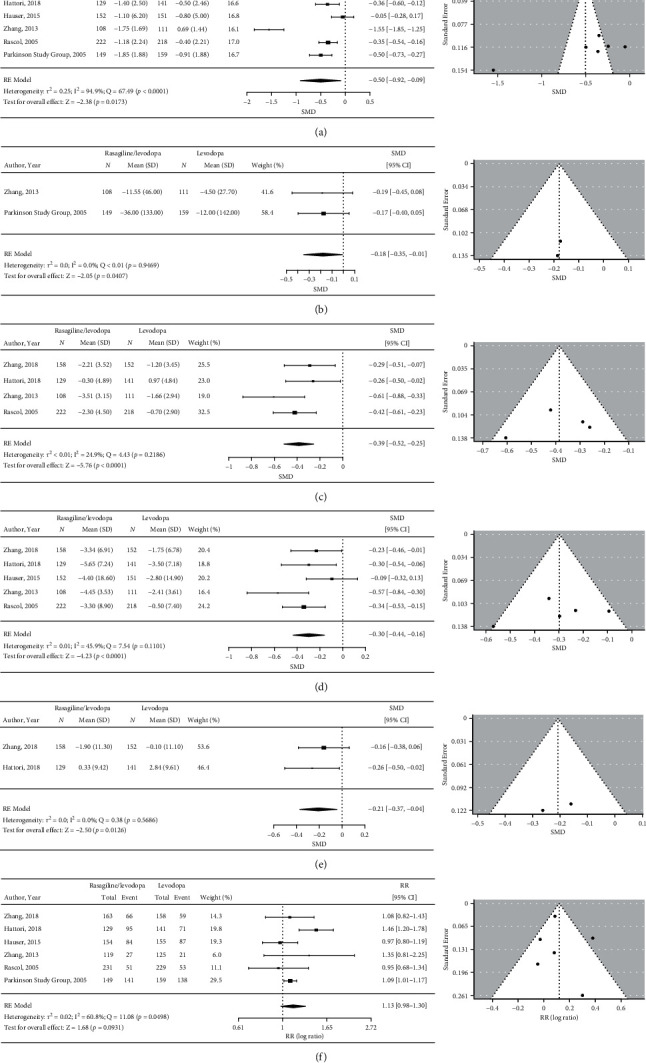
Forest plots and funnel plots for treatment effects of rasagiline/levodopa combination therapy versus levodopa monotherapy on (a) wearing-off time (hours), (b) levodopa dosage (mg/day), (c) UPDRS/MDS-UPDRS II score, (d) UPDRS/MDS-UPDRS III score, (e) PDQ-39 summary index score, and (f) incidence of TEAEs. CI, confidence interval; MDS-UPDRS, Movement Disorder Society-Unified Parkinson's Disease Rating Scale; PDQ-39, Parkinson's Disease Questionnaire; RE, random effect; RR, risk ratio; SD, standard deviation; SMD, standardized mean difference; TEAE, treatment-emergent adverse event; UPDRS, Unified Parkinson's Disease Rating Scale.

All six studies assessed the change in wearing-off time [17, 18, 24, 30–32]. A statistically significant reduction in wearing-off time was observed in patients treated with rasagiline/levodopa compared with placebo/levodopa (SMD = −0.50, 95% CI:−0.92 to −0.09, *p* = 0.017; Figure 3(a); MD = −1.03, 95% CI: −1.65 to −0.40). There was extreme heterogeneity between the studies (I^2^ = 94.9%), with Egger's test showing significant publication bias (Egger's test: *p* = 0.092; Begg's test: *p* = 0.272) and an asymmetric funnel plot. A consistent result was observed in the sensitivity analysis, in which the study considered at high risk of bias [31] was excluded (Figure S1(a)). In the sensitivity analysis, lower heterogeneity (I^2^ = 52.1%) was observed; the funnel plot showed symmetry and there was also no evidence of publication bias with Egger's test (*p* = 0.892) or Begg's test (*p* = 1).

#### 3.3.2. Treatment Effect of Rasagiline on Levodopa Dosage

There were two studies that assessed the change in levodopa dosage [17, 31]. The analysis revealed that the reduction in levodopa dosage was statistically significant in patients treated with rasagiline/levodopa versus placebo/levodopa (SMD = −0.18, 95% CI: −0.35 to –0.01, *p* = 0.041; Figure 3(b); MD = −25.56, 95% CI: −49.70 to −1.42). No heterogeneity was observed between the studies (I^2^ = 0.0%). Furthermore, publication bias was not evaluated because only two studies were included in this analysis.

#### 3.3.3. Treatment Effect of Rasagiline on UPDRS/MDS-UPDRS II Score

There were four studies that assessed the change in UPDRS/MDS-UPDRS II score [18, 24, 30, 31]. A statistically significant reduction in UPDRS/MDS-UPDRS II score was observed in patients treated with rasagiline/levodopa compared with placebo/levodopa (SMD = –0.39, 95% CI: –0.52 to –0.25, *p* < 0.0001; Figure 3(c); MD = –1.13, 95% CI: −1.51 to −0.73). There was low heterogeneity among the studies (I^2^ = 24.9%). Both Egger's test (*p* = 0.757) and Begg's test (*p* = 1) showed no evidence of publication bias; the funnel plot also showed symmetry. Furthermore, the results of the sensitivity analysis excluding the study with a high risk of bias [31] were consistent with the results of the primary analysis (Figure S1(b)). In the sensitivity analysis, heterogeneity was not observed (I^2^ = 0.0%). No significant publication bias was observed with Begg's test (“next to” *p* = 0.333), and the funnel plot showed symmetry, although a significant publication bias was observed with Egger's test (*p* = 0.083).

#### 3.3.4. Treatment Effect of Rasagiline on UPDRS/MDS-UPDRS III Score

There were five studies that assessed the change in UPDRS/MDS-UPDRS III score [18, 24, 30–32]. In the primary analysis, a statistically significant reduction in UPDRS/MDS-UPDRS III score was observed in patients treated with rasagiline/levodopa compared with placebo/levodopa (SMD = –0.30, 95% CI: −0.44 to −0.16, *p* < 0.0001; Figure 3(d); MD = −2.22, 95% CI: −3.26 to −1.18). Although heterogeneity among the studies was moderate (I^2^ = 45.9%), there was no evidence of publication bias (Egger's test: *p* = 0.585, Begg's test: *p* = 0.817), with the funnel plot showing symmetry. The results of the sensitivity analysis excluding the study with a high risk of bias [31] were consistent with the results of the primary analysis (Figure S1(c)) but with lower heterogeneity (I^2^ = 1.9%). The funnel plot remained symmetric, and Egger's test (*p* = 0.488) and Begg's test (*p* = 0.750) also suggested nonsignificant publication bias.

#### 3.3.5. Treatment Effect of Rasagiline on PDQ-39 Summary Index Score

There were two studies that assessed the change in PDQ-39 summary index score [24, 30]. The primary analysis revealed that the reduction in PDQ-39 summary index score was statistically significant in patients treated with rasagiline/levodopa compared with those treated with placebo/levodopa (SMD = −0.21, 95% CI: −0.37 to –0.04, *p* = 0.013; Figure 3(e); MD = –2.33, 95% CI: −4.11 to −0.44). In this analysis, no heterogeneity was observed between the studies (I^2^ = 0.0%). However, publication bias was not evaluated because only two studies were included in this analysis.

### 3.4. Safety

All six studies reported the overall incidence of TEAEs. The primary analysis revealed that there was no statistically significant difference in the incidence of TEAEs between patients treated with rasagiline/levodopa compared with placebo/levodopa (RR = 1.13, 95% CI: 0.98–1.30, *p* = 0.093; Figure 3(f)). Heterogeneity was moderate to high among the studies (I^2^ = 60.8%). Although the funnel plot showed a mild asymmetry, both Egger's test (*p* = 0.757) and Begg's test (*p* = 1) showed no evidence of publication bias. Furthermore, a sensitivity analysis excluding the study with a high risk of bias [31] showed results consistent with the primary analysis (Figure S1(d)). However, heterogeneity was still moderate to high (I^2^ = 67.2%). In the sensitivity analysis, the funnel plot also still showed a mild asymmetry, but no publication bias was evident (Egger's test: *p* = 0.936; Begg's test: *p* = 0.817).

## 4. Discussion

This systematic review and meta-analysis evaluated the efficacy and safety of rasagiline in levodopa-treated patients with PD experiencing motor fluctuations. Previous RCTs, including TEMPO [15], ADAGIO [16], PRESTO [17], and LARGO [18], have demonstrated the efficacy and safety of rasagiline as monotherapy and as an adjunct to levodopa. This was the first review to include studies that used the revised MDS-UPDRS rating scale and to integrate it with the conventional UPDRS and the first to include the PDQ-39 summary index score in the analysis. In this meta-analysis, significant improvements in wearing-off time, levodopa dosage, UPDRS/MDS-UPDRS II and III scores, and PDQ-39 summary index score were observed in patients treated with rasagiline/levodopa combination therapy compared with those treated with placebo/levodopa, although heterogeneity and publication bias were present for some outcomes. Conversely, there was no difference in the incidence of TEAEs between the treatment groups. Therefore, this review further confirmed the efficacy and safety of rasagiline as an adjunct therapy in levodopa-treated patients with PD experiencing motor fluctuations, which was consistent among various populations including those from the US, Europe, and Japan.

The MDS-UPDRS is currently the most popular rating scale in research settings, addressing problematic areas of the conventional UPDRS. The MDS-UPDRS resolved ambiguities in language, structural inconsistency, and inconsistency with the questions, with numerical options not always reflecting the same level of dysfunction present in the original UPDRS [19]. The MDS-UPDRS also enabled the detection of small changes in early disease and the differentiation of relatively mild impairments and disabilities [19, 20]. Furthermore, retaining the four-part structure of the UPDRS [20], Part I now focuses on nonmotor aspects of the disease, and Part II of the MDS-UPDRS was renamed “Motor Experiences of Daily Living,” with the conceptual construct focusing on the impact of the motor symptoms rather than the presence of symptoms [19, 20]. Despite these revisions, Parts II and III of the MDS-UPDRS highly correlate with the corresponding parts of the original UPDRS [20, 33, 34], and therefore the assumption of the Hedge's *g* is met, justifying its use to integrate the two scales and calculate the SMD.

This current meta-analysis was the first to include this revised rating scale (MDS-UPDRS). In this analysis, rasagiline/levodopa combination therapy was associated with significant improvements in UPDRS/MDS-UPDRS II score (SMD = –0.39, 95% CI: –0.52 to –0.25, *p* < 0.0001) and UPDRS/MDS-UPDRS III score (SMD = –0.30, 95% CI: –0.44 to –0.16, *p* < 0.0001) compared with levodopa monotherapy, which was consistent with the results observed in previous meta-analyses that only included the conventional UPDRS. In a meta-analysis of 14 RCTs, rasagiline/levodopa combination therapy significantly improved the UPDRS II score (*n* = 1533, nine RCTs, SMD = –0.59, 95% CI: –0.79 to –0.39, *p* < 0.00001) and UPDRS III score (*n* = 2111, 12 RCTs, SMD = –0.50, 95% CI: −0.70 to −0.30, *p* < 0.00001) in patients with idiopathic PD of any stage [35]. An improvement in MDS-UPDRS III of >3.25 points has been shown to represent minimal clinically important change based on clinician-rated Clinical Global Impression-Improvement scores [36]. Although not directly comparable, this study reported that the MDS-UPDRS III score was improved by 2.22 points with rasagiline/levodopa combination therapy versus levodopa monotherapy; this was similar to a meta-analysis that evaluated the efficacy of rasagiline 0.5–1 mg/day in levodopa-treated patients with idiopathic PD (*n* = 712, two RCTs), which reported an MD of –2.91 (95% CI: −4.02 to −1.80) points, demonstrating that UPDRS III score was significantly improved with rasagiline 1 mg/day compared with placebo (*p* < 0.00001) [37]. Therefore, the results from this analysis further confirmed that even when assessed with the revised MDS-UPDRS, rasagiline/levodopa combination therapy is effective in improving motor functions and motor aspects of daily activities compared with levodopa monotherapy in PD with motor fluctuations. However, these results should be interpreted with caution; although MDS-UPDRS highly correlates with UPDRS, and the results of previous studies that used UPDRS scores as an outcome are important and cannot be ignored, there may be limitations in integrating the results of studies that used two scales with different wording, structure, and item responses.

In this study, although moderate to high heterogeneity was observed even in the sensitivity analysis, wearing-off time was significantly reduced in patients with rasagiline/levodopa combination therapy versus levodopa monotherapy (SMD = −0.50, 95% CI: −0.92 to −0.09, *p*=0.017). The MD in wearing-off time between rasagiline/levodopa combination therapy and levodopa monotherapy was −1.03 (95% CI: −1.65 to −0.40) hours, which was similar to the results observed in a previous meta-analysis by Cai et al. (*n* = 712, two RCTs, MD = –0.86, 95% CI: −1.15 to −0.56, *p* < 0.00001) [37]. Importantly, given that a reduction in wearing-off time of 1 hour may be considered a clinically meaningful change [38], these results collectively indicate that rasagiline/levodopa combination therapy is effective in reducing wearing-off time compared with levodopa monotherapy in patients with PD with motor fluctuations.

PDQ-39 summary index score is an important outcome measure that enables disease assessment from the patients' perspectives, which then allows shared decision-making by physicians and patients in the management of PD. A postal survey of the Parkinson's Disease Society members in the UK reported that minimally important differences varied across the dimensions of the PDQ-39 [39]. Another study reported that the minimal clinically important difference estimate for the PDQ-39 summary index score was 4.72 points for improvement, which was calculated using the Patient Global Impression-Improvement as an anchor [40]. Additionally, previous studies have shown that PDQ-39 scores are significantly associated with MDS-UPDRS II and III scores and wearing-off time [41, 42]. In particular, there is increasing evidence that PDQ-39 scores are more tightly associated with the MDS-UPDRS II score than with the MDS-UPDRS III score and that impairments in motor activities of daily living significantly contribute to poorer health-related QOL [42–44]. Similar to the results observed for other efficacy outcomes, in this current meta-analysis, rasagiline/levodopa combination therapy significantly improved the PDQ-39 summary index score by 2.33 points compared with levodopa monotherapy. Therefore, these findings further indicated that QOL correlates with other efficacy outcomes, including motor experiences of daily living and motor functions. This analysis provided valuable results that suggest that rasagiline/levodopa combination therapy may be superior in improving health-related QOL compared with levodopa monotherapy in PD with motor fluctuations. As there were only two studies included for the analysis of PDQ-39, future RCTs to establish the effect of rasagiline on PDQ-39 score are anticipated.

Although moderate to high heterogeneity was observed, this current analysis showed that there was no significant difference in the incidence of TEAEs between rasagiline/levodopa combination therapy versus levodopa monotherapy. This was also consistent with the results from previous meta-analyses [35, 37]. Therefore, these results further support the safety of rasagiline plus levodopa in patients with PD experiencing motor fluctuations among various populations, including in Japanese patients.

The main strength of this study was the comprehensiveness of the systematic literature search and meta-analysis. We included studies that used the MDS-UPDRS score and summarized the results by integrating it with the conventional UPDRS used in other studies. We also assessed a variety of outcomes, including the revised UPDRS/MDS-UPDRS II score and PDQ-39 summary index score, which are new outcomes that evaluate the effect of rasagiline on motor experiences of daily living and QOL. However, this meta-analysis was limited by the inclusion of publications written in English only and of one study that did not use ITT analysis, which was therefore considered to have a high risk of bias. Publication bias was present in the primary analysis for change in wearing-off time; however, there was no evidence of publication bias in the sensitivity analysis, which excluded the one study with a high risk of bias. There were also high levels of heterogeneity among the studies for some analyzed outcomes; again, the exclusion of the study that did not use ITT analysis reduced the heterogeneity. Furthermore, SMD was used as the main measure to estimate the treatment effects; however, SMD may not be easily interpretable to determine the clinical relevance of the results. The MD was calculated as a reference by backtransforming the SMD; however, this potentially may have yielded misleading effect sizes for each outcome [45]. There was a limited number of studies analyzed for some outcomes, such as a change in levodopa dosage; therefore, future studies are required to further analyze and confirm the effects of rasagiline on these outcomes.

## 5. Conclusions

This meta-analysis demonstrated that rasagiline plus levodopa is superior in improving UPDRS/MDS-UPDRS II score, UPDRS/MDS-UPDRS III score, PDQ-39 score, and wearing-off time compared with levodopa monotherapy in patients with PD experiencing motor fluctuations. This meta-analysis also confirmed the safety profile of rasagiline plus levodopa, with a similar incidence of TEAEs compared with levodopa monotherapy.

## Figures and Tables

**Table 1:** Characteristics of studies included.

Study	Study duration, weeks	*N*	Male, *n* (%)	Age, years^a^	PD duration, years^a^	Baseline levodopa dosage, mg/day^a^
Zhang, 2018 [30]	16	R/L: 163 L: 158	R/L: 103 (63.2) L: 109 (69.0)	R/L: 62.7 L: 61.7	R/L: 7.4^b^ L: 7.1^b^	R/L: 501.0^b^ L: 550.0^b^

Hattori, 2018 [24]	26	R/L: 129 L: 141	R/L: 46 (35.7) L: 53 (37.6)	R/L: 65.8 L: 66.3	R/L: 9.5 L: 8.9	R/L: 421.0 L: 399.3

Hauser, 2015 [32]	12	R/L: 154 L: 155	R/L: 95 (61.7) L: 78 (50.3)	R/L: 63.6 L: 63.0	R/L: 8.3^c^ L: 8.2^c^	R/L: 800.0^c^ L: 650.0^c^

Zhang, 2013 [31]	12	R/L: 119 L: 125	R/L: 64 (53.8) L: 67 (53.6)	R/L: 61.6 L: 61.6	R/L: 5.6 L: 5.4	R/L: 515.0 L: 521.0

Rascol, 2005 [18]	18	R/L: 231 L: 229	R/L: 154 (66.7) L: 132 (57.6)	R/L: 63.9 L: 64.8	R/L: 8.7 L: 8.8	R/L: 722.0 L: 697.0^d^

Parkinson Study Group, 2005 [17]	26	R/L: 149 L: 159	R/L: 99 (66.4) L: 104 (65.4)	R/L: 62. L: 64.5	R/L: 8.8 L: 9.7	R/L: 815.0 L: 821.0

^a^All values are means unless otherwise indicated ^b^Not explicitly reported in publication but assumed to be mean ^c^Median. ^d^Not explicitly reported in publication but assumed to be daily levodopa dose (mg/day). *L*, levodopa; PD, Parkinson's disease; R/L, rasagiline/levodopa combination therapy.

## Data Availability

The datasets used and/or analyzed during the current study are available from the corresponding author on reasonable request.

## Abbreviations

CI: Confidence interval
ITT: Intention to treat
MD: Mean difference
MDS: Movement Disorder Society
PD: Parkinson's disease
PDQ-39: Parkinson's Disease Questionnaire
QOL: Quality of life
RCT: Randomized controlled trial
RR: Risk ratio
SD: Standard Deviation
SMD: Standard Mean Difference
TEAE: Treatment-emergent adverse event
UPDRS: Unified Parkinson's Disease Rating Scale.

## Supplementary Materials

Supplementary Materials Table S1: search strategy for each database. Figure S1: sensitivity analysis for rasagiline/levodopa combination therapy versus levodopa monotherapy on (A) wearing-off time (hours), (B) UPDRS/MDS-UPDRS II, (C) UPDRS/MDS-UPDRS III, and (D) incidence of TEAEs.
