# Supplementary material for: Rasagiline as Adjunct to Levodopa for Treatment of Parkinson's Disease: A Systematic Review and Meta-Analysis
Source: Parkinsons Dis. 2022 Aug 30;2022:4216452. doi: 10.1155/2022/4216452 (PMC9448622; doi:10.1155/2022/4216452)

Supplementary materials

Table S1: Search strategy for each database.

PubMed Literature Search Strategy: Search conducted on January 18, 2021.

| # | Strategy key terms | Results, *n* |
| --- | --- | --- |
| 1 | "parkinson*"[TW] or “Parkinsonian Disorders”[MESH] | 101,585 |
| 2 | “rasagiline”[TW] or “azilect”[TW] or “agilect”[TW] or “AGN-1135”[TW] or “AGN1135”[TW] or “TVP-101”[TW] or “TVP101”[TW] or “TVP-1012”[TW] or “TVP1012”[TW] or "rasagiline" [Supplementary Concept] | 578 |
| 3 | “l-dopa”[TW] or “ldopa”[TW] or “levodopa”[TW] | 18,408 |
| 4 | "Randomized Controlled Trial"[PT] or “Controlled Clinical Trial”[PT] or “randomized”[TIAB] or “placebo”[TIAB] or “clinical trials as topic”[MESH: noexp] or “randomly”[TIAB] or “trial”[TI] NOT (“animals”[MH] NOT “humans“[MH]) | 1,019,697 |
| #5 | #1 and #2 and #3 and #4 | 71 |

Cochrane Library Literature Search Strategy: Search conducted on January 18, 2021.

| # | Strategy key terms | Results, *n* |
| --- | --- | --- |
| #1 | MeSH descriptor: [Parkinson Disease] explode all trees | 4233 |
| #2 | (Parkinson*): ti; ab; kw | 10,857 |
| #3 | #1 or #2 | 10,857 |
| #4 | rasagiline or azilect or agilect or AGN-1135 or AGN1135 or TVP-101 or TVP101 or TVP-1012 or TVP1012: ti; ab; kw | 242 |
| #5 | l-dopa or ldopa or levodopa: ti; ab; kw | 3705 |
| #6 | #3 and #4 and #5 (in Trials) | 86 |

EMBASE Library (via ProQuest) Search Strategy: Search conducted on January 19, 2021.

| # | Strategy key terms | Results, *n* |
| --- | --- | --- |
| 1 | ti,ab,su(Parkinson*) | 215,355 |
| 2 | ti,ab,su(rasagiline or azilect or agilect or AGN-1135 or AGN1135 or TVP-101 or TVP101 or TVP-1012 or TVP1012) | 2794 |
| 3 | i,ab,su(l-dopa or ldopa or levodopa) | 59,445 |
| 4 | #2 and #3 | 1645 |
| 5 | #1 and #4 | 1614 |
| 6 | ti,ab(random*) | 1,618,038 |
| 7 | ti,ab,su(placebo) | 467,464 |
| 8 | ti,ab("double-blind*") | 216,069 |
| 9 | #8 or #9 or #10 | 1,877,563 |
| 10 | #5 and #9 | 407 |

Web of Science Search Strategy: Search conducted on January 18, 2021.

| # | Strategy key terms | Results, *n* |
| --- | --- | --- |
| 1 | Topic=(clin*) OR Title=(clin*) | 4,140,887 |
| 2 | Topic=(trial*) OR Title=(trial*) | 1,662,370 |
| 3 | #1 and #2 | 751,181 |
| 4 | TS=(singl* OR Doubl* OR Tripl* OR Trebl*) OR TI=(singl* OR Doubl* OR Tripl* OR Trebl*) | 4,768,801 |
| 5 | TS=(mask* OR blind*) OR TI=(mask* OR blind*) | 669,640 |
| 6 | #4 and #5 | 347,250 |
| 7 | TS=(allocate* OR assign*) OR TI=(allocate* OR assign*) | 717,981 |
| 8 | TS=random* OR TI=random* | 1,957,768 |
| 9 | #7 and #8 | 200,228 |
| 10 | #3 or #6 or #9 | 1,126,658 |
| 11 | TS=(rasagiline or azilect or agilect or AGN-1135 or AGN1135 or TVP-101 or TVP101 or TVP-1012 or TVP1012) | 1144 |
| 12 | TS=(l-dopa or ldopa or levodopa) | 29,049 |
| 13 | #11 and #12 | 328 |
| 14 | TS=(parkinson*) | 186,837 |
| 15 | #10 and #13 and #14 | 212 |

Figure S1: Sensitivity analysis for rasagiline/levodopa combination therapy versus levodopa monotherapy on (A) wearing-off time (hours), (B) UPDRS/MDS-UPDRS II, (C) UPDRS/MDS-UPDRS III, and (D) incidence of TEAEs. CI, confidence interval; MDS-UPDRS, Movement Disorder Society-Unified Parkinson’s Disease Rating Scale; RE, random effect; RR, risk ratio; SD, standard deviation; SMD, standardized mean difference; TEAE, treatment-emergent adverse event; UPDRS, Unified Parkinson’s Disease Rating Scale.


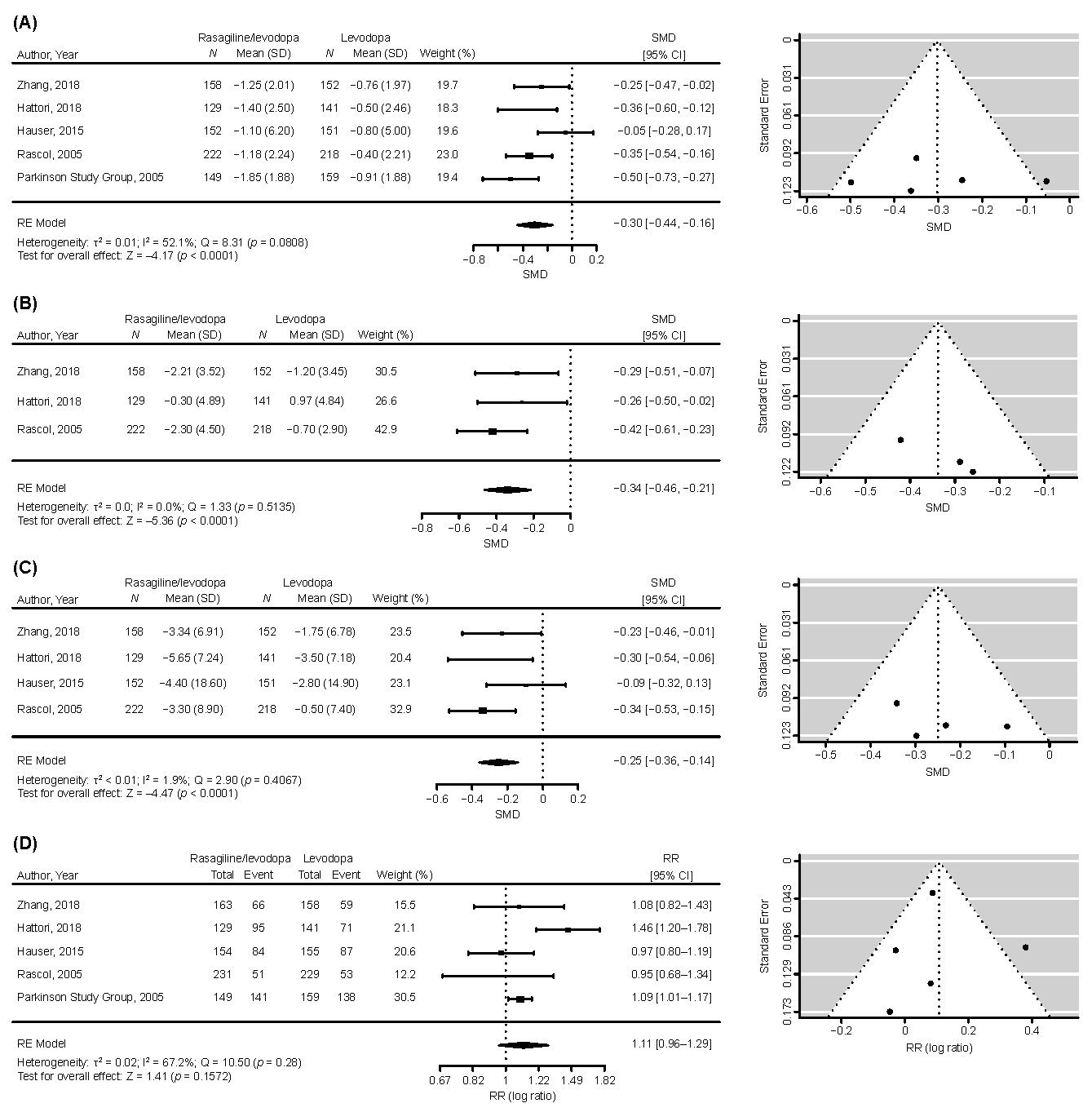

Supplement: Supplementary Materials — Table S1: search strategy for each database. Figure S1: sensitivity analysis for rasagiline/levodopa combination therapy versus levodopa monotherapy on (A) wearing-off time (hours), (B) UPDRS/MDS-UPDRS II, (C) UPDRS/MDS-UPDRS III, and (D) incidence of TEAEs. [file 4216452.f1.zip › 4216452.f1/PD SR meta-analysis MS_21Feb22_V8.0_Final_Supplementary.docx]
